# Hydroclimatic drivers of highly seasonal leptospirosis incidence suggest prominent soil reservoir of pathogenic *Leptospira* spp. in rural western China

**DOI:** 10.1371/journal.pntd.0007968

**Published:** 2019-12-26

**Authors:** Karina Cucchi, Runyou Liu, Philip A. Collender, Qu Cheng, Charles Li, Christopher M. Hoover, Howard H. Chang, Song Liang, Changhong Yang, Justin V. Remais

**Affiliations:** 1 University of California, Berkeley, Berkeley, California, United States of America; 2 Sichuan Center for Disease Control and Prevention, Chengdu, Sichuan, China; 3 Emory University, Atlanta, Georgia, United States of America; 4 University of Florida, Gainesville, Florida, United States of America; Yale University, UNITED STATES

## Abstract

Climate exerts complex influences on leptospirosis transmission, affecting human behavior, zoonotic host population dynamics, and survival of the pathogen in the environment. Here, we describe the spatiotemporal distribution of leptospirosis incidence reported to China’s National Infectious Disease Surveillance System from 2004–2014 in an endemic region in western China, and employ distributed lag models at annual and sub-annual scales to analyze its association with hydroclimatic risk factors and explore evidence for the potential role of a soil reservoir in the transmission of *Leptospira* spp. More than 97% of the 2,934 reported leptospirosis cases occurred during the harvest season between August and October, and most commonly affected farmers (83%). Using a distributed lag Poisson regression framework, we characterized incidence rate ratios (IRRs) associated with interquartile range increases in precipitation of 3.45 (95% confidence interval 2.57–4.64) over 0-1-year lags, and 1.90 (1.18–3.06) over 0-15-week lags. Adjusting for soil moisture decreased IRRs for precipitation at both timescales (yearly adjusted IRR: 1.05, 0.74–1.49; weekly adjusted IRR: 1.36, 0.72–2.57), suggesting precipitation effects may be mediated through soil moisture. Increased soil moisture was positively associated with leptospirosis at both timescales, suggesting that the survival of pathogenic *Leptospira* spp. in moist soils may be a critical control on harvest-associated leptospirosis transmission in the study region. These results support the hypothesis that soils may serve as an environmental reservoir and may play a significant yet underrecognized role in leptospirosis transmission.

## Author summary

Leptospirosis is among the leading causes of morbidity from zoonotic infections worldwide, affecting populations that are exposed to contaminated water. The disease is caused by *Leptospira* spp. bacteria, which are transmitted to humans either through direct contact with infected animals, or indirectly through the environment. Climatic conditions can influence transmission by altering human exposure, animal host population dynamics, and environmental conditions that allow *Leptospira* spp. to persist in the environment (e.g., moist environments, warm temperatures). Here, we investigated the spatiotemporal distribution of leptospirosis cases in a rural setting in western China and estimated the association between hydroclimatic conditions and leptospirosis incidence. We found that incidence of leptospirosis—especially high amongst farmers—may be associated with rice harvest, and modulated by prior bacterial accumulation within the soil under moist conditions. These results corroborate previous findings that soils may be underrecognized environmental reservoirs of pathogenic *Leptospira* spp., and that their role in explaining leptospirosis incidence should be considered when developing prevention programs.

## Introduction

Leptospirosis is a reemerging infectious zoonosis caused by pathogenic bacteria from the genus *Leptospira*. The disease is among the leading zoonotic causes of morbidity worldwide, infecting an estimated 1.03 million people, and causing 58,900 deaths per year associated with severe manifestations of the infection such as pulmonary hemorrhage syndrome and Weil’s disease [1,2]. The infection is transmitted to humans by contact with contaminated water, or by direct contact with infected animals. The onset of symptoms averages 7 to 12 days after exposure, although the incubation period can extend from 3 days to as long as a month [3]. Pathogenic leptospires persist through continuous enzootic circulation among mammalian species that serve as reservoirs for transmission [4,5]. Rodents are particularly important asymptomatic carriers that can shed contaminated urine in the environment for their entire lifespan [2]; they are considered a major reservoir host for human leptospirosis [6].

Environmental and climatic conditions may influence leptospirosis risk by affecting the distribution and abundance of mammalian hosts, pathogen survival in water and soil, and human exposure to the bacteria. Rainfall, in particular, has been identified as a key environmental driver in numerous observational studies [7,8].

Several causal pathways have been proposed linking variations in rainfall to leptospirosis risk, involving different intermediate environmental and ecological processes and operating on different timescales (Table 1). As rainfall hits the land surface, it is either stored in the subsurface as soil moisture, or is lost from the soil reservoir to evapotranspiration or surface runoff [23,24]. Changes in water availability in the soil and/or surface runoff can influence leptospirosis transmission. For example, rodent abundance has been found to be influenced by precipitation during the preceding wet season, as changes in soil moisture impact primary production and food availability, resulting in higher rodent populations the following year and having implications for leptospirosis transmission [9]. There is considerable uncertainty regarding the timescale of pathogenic leptospire survival following excretion in the environment, with estimates ranging from weeks to a year, depending on soil moisture and temperature conditions, among other factors [14–18]. Over short timescales (weeks to months), extreme precipitation leading to runoff and flooding have a well-documented association with leptospirosis incidence in human populations, which may be due to increased contact rates with contaminated water, or more frequent contacts between humans and animal hosts driven into confined areas following inundation [19,20]. Large-scale hydrological simulations allow the estimation of rainfall partitioning between soil moisture and surface runoff at spatio-temporal scales relevant to environmental transmission pathways, yet no study to date has examined estimated soil moisture or surface runoff as mediating variables to investigate the pathways that link rainfall to leptospirosis incidence.

**Table 1:** Summary of pathways linking rainfall to leptospirosis risk, with timescales of estimated associations.

Environmental transmission pathway	Hydrological mediator	Putative timescale of association with reported cases	Supporting references
Precipitation increases soil moisture, influencing primary production, rodent abundance, and rodent infection rates	Soil moisture	Annual to multi-annual	[9–13]
Precipitation increases soil moisture, which, in concert with favorable temperatures, favors survival and accumulation of *Leptospira* spp. in the soil	Soil moisture	Weeks to months	[14–18]
Extreme rainfall results in flooding, driving humans and rodents into closer proximity and bringing humans into contact with water contaminated by *Leptospira* spp. flushed from soils	Surface runoff	1–4 weeks of incubation time after acute exposure	[3,19–22]

Leptospirosis outbreaks are frequent in China, but the risk factors for transmission are poorly understood [25]. Incidence occurs seasonally in well-defined cycles characterized by high incidence in summer and early autumn [26]. An ecological niche analysis of 2,741 leptospirosis cases occurring throughout much of China between 2010 and 2014 identified mean annual temperature and annual precipitation as the factors most strongly associated with leptospirosis presence among nine candidate environmental and socioeconomic variables [25]. However, temporal aspects of leptospirosis occurrence, and the role of the various mechanisms potentially underlying these coarse spatial associations remain unexplored. Individual outbreaks—such as the outbreak in Lezhi County, Sichuan, in 2010—are often thought to be linked to extreme precipitation [21], though quantitative analyses supporting this hypothesis are lacking. While leptospirosis incidence in China has declined since 2005, substantial burden remains, and rapid environmental and social changes are likely to impact the risk of reemergence in urban and peri-urban areas [27]. A deeper understanding of the relative importance of risk factors—including environmental conditions that support transmission—can aid in anticipating and mitigating the burden of leptospirosis in China, and in settings with similar epidemiological and environmental conditions.

In this study, we explore high-resolution spatiotemporal patterns of leptospirosis in Sichuan, China (pop: 87 mil), from 2004 to 2014, and examine their association with hydroclimatic risk factors at inter- and intra-annual timescales. We explore the mechanisms that underlie associations between precipitation and incidence, using data derived from a large-scale land surface hydrological model of the region to analyze the mediation of precipitation effects through soil moisture and surface runoff.

## Materials and methods

### Study area

With an area of 485,000 km^2^ and a population of more than 80 million in 2010, Sichuan is amongst the largest and most populated provinces in China. Environmental and climatic conditions in Sichuan vary most sharply between its western and eastern regions: western regions consist of high mountains and plateaus, and eastern regions, where most of the population is located, consist of low elevation fertile plains. Sichuan is divided into 21 prefectures, further divided into counties (181 counties in total), ranging in population from 2,600 to 159,000 people in 2010 (median population of 42,300). Sichuan’s economy heavily relies on agriculture, with 70.5% of the population reporting agriculture as their occupation as of 2014, and rice being the dominant crop [28]. Sichuan has a subtropical monsoon climate, with a mean temperature of 16–18°C and an annual average precipitation of 1,000–1,200 mm, which occurs mostly in the wet season (May-October). Rice harvest timing ranges between mid-August to mid-September over a geographical gradient spanning South to North, and from low to high elevation (Quanzhong Ge, Sichuan Department of Agriculture, pers. comm.). Climatic, hydrological, and occupational factors in the region may favor leptospirosis transmission, potentially explaining why Sichuan is one of several provinces in China with high leptospirosis risk, and for which surveillance and control programs are a priority [25,26].

### Health data and case definition

China’s National Infectious Disease Reporting System (NIDRS) was queried for leptospirosis cases reported between 2004 and 2014. The NIDRS is a country-wide online disease reporting network mandating submission of case reports within 24 hours of diagnosis of 39 reportable infectious diseases, including leptospirosis, at any clinic or hospital in China [29]. Cases were diagnosed following official Chinese protocols [30,31]. Suspected cases were defined as having contacted contaminated water 1–30 days before the symptom onset, and the presence of fever, muscle ache, or fatigue. Clinical cases must also have conjunctival suffusion, gastrocnemius pain, or swelling of lymph nodes, while laboratory confirmed cases were defined as having a positive test result for either darkfield microscopy, culture, polymerase chain reaction (PCR), Microscopic Agglutination Test (MAT), or indirect enzyme-linked immunosorbent assay (ELISA). All three classifications of cases (suspected, clinically diagnosed, and confirmed) were included in this study. Gender, age, occupation, residence location, date of diagnosis, and date of death (if applicable) were extracted for each case.

### Ethics statement

This study utilized patient medical data. This study was approved by the Committee for Protection of Human Subjects at the University of California, Berkeley, and all personally-identifiable data were removed from the dataset prior to analysis.

### Hydroclimatic data

Hydroclimatic data were retrieved from the China hydrologic dataset developed elsewhere [32], consisting of long-term, gridded meteorological data from ground monitoring stations located across China, with good gauge coverage of the Sichuan province [33]. In the prior work [32], meteorological data were used as forcing variables for the Variable Infiltration Capacity (VIC) hydrological model, in order to simulate land surface hydrological conditions from 1952 to 2012. The VIC model is a widely used, large-scale hydrological model that represents water and heat fluxes between the land and the atmosphere, as well as water and energy balances at the land surface [34]. In its prior application [32], VIC was calibrated based on observed monthly streamflow at 15 hydrological stations, and validated against soil moisture at 43 measurement stations located across China. Both model inputs and outputs to the model were available for the present project at a 0.25° spatial resolution (~28km) and at a daily temporal resolution (Table 2). We retrieved daily gridded precipitation, surface runoff, soil moisture, and temperature data from the database [32] for Sichuan province, over the time period overlapping leptospirosis case data (i.e., 2003–2012 accounting for up to one-year lag of hydroclimatic influences), and aggregated these data to county-year and county-week resolutions.

**Table 2:** Summary of hydroclimatic variables used in regression analyses of human leptospirosis infections in Sichuan, with abbreviations.

Variable	Symbol	Description	25th and 75th percentile*	Lags considered in regression models
Precipitation	P	Mean daily precipitation	Yearly 2.52–3.19 mm Weekly 1.07–5.43 mm	Yearly 0–1 years Weekly 0–15 weeks
Minimum temperature	T_min_	Mean minimum daily temperature	Yearly 12.03–14.71°C Weekly 14.50–21.25°C	Yearly 0 years Weekly 2 weeks
Runoff	Q	Mean surface runoff rate	Yearly 0.62–0.98 mm/s Weekly 0.146–1.378 mm/s	Yearly 0 years Weekly 0–15 weeks
Soil moisture	θ	Mean water content in the top 10cm of the soil	Yearly 26.00–27.19 mm Weekly 26.08–29.47 mm	Yearly 0–1 years Weekly 0–15 weeks

*25^th^ and 75^th^ percentile of values observed across all years / weeks and counties.

### Spatiotemporal descriptive analyses

Annual and weekly incidence rates were estimated at the county level using population data for years 2005, 2008, 2010, and 2014 [28], with population sizes at intermediate years estimated by linear interpolation. We mapped county-level yearly leptospirosis incidence and derived timeseries of yearly and weekly leptospirosis incidence. Exploratory analyses were conducted using the R statistical software [35], and results were plotted using the ggplot2 library [36]. We used spatial Poisson scan statistics to detect high incidence rate clusters over the full study period, and ran spatiotemporal scan statistics to detect yearly clusters, testing the membership of individual counties in the detected clusters using Oliveira’s F statistic, as implemented by SaTScan [37–39]. To explore the seasonality of leptospirosis incidence, we fit time series of weekly case counts to a cubic B-spline wavelet, used to achieve optimal time-frequency localization [40]. Amplitude, timing and duration of the spline were estimated using least squares. In order to explore spatiotemporal variation in leptospirosis seasonality, we separately fitted cubic B-spline wavelets to weekly counts of cases in each 0.5° latitude band and each year.

### Analysis of hydroclimatic risk factors

**Fig 1:**
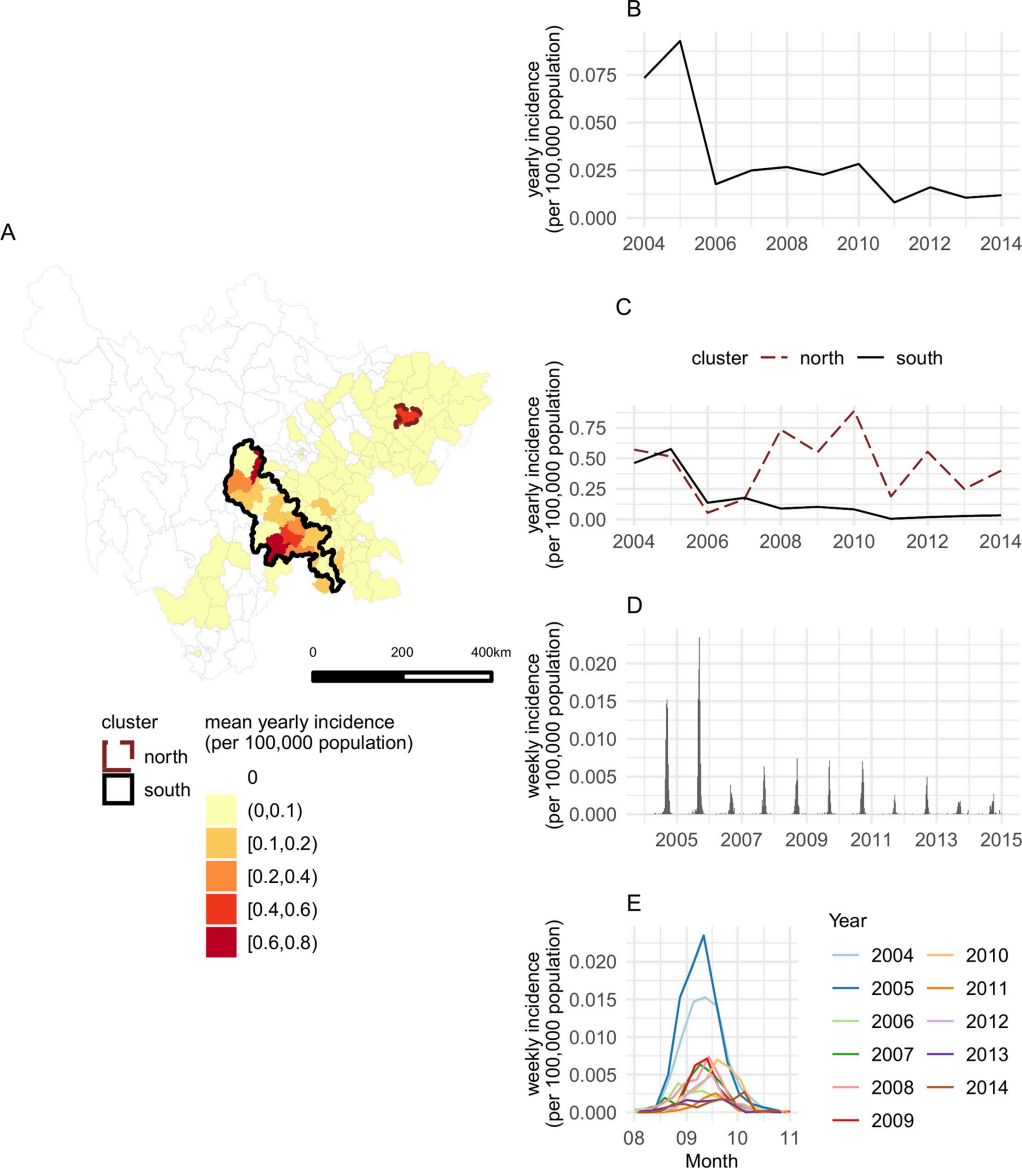
Spatial and temporal distribution of leptospirosis incidence in Sichuan, 2004–2014. A: Mean yearly leptospirosis incidence by county, aggregated over the study period. This map was generated using data provided by Sichuan CDC for the purpose of this study, and plotted in R statistical software using ggplot2. B: Time series of yearly incidence in Sichuan. C: Time series of yearly incidence within the 2 high-risk clusters. D: Time series of weekly incidence in Sichuan. E: Time series of weekly incidence in Sichuan, with a focus on seasonal peaks.

In the interest of investigating mechanisms of leptospirosis transmission driven by precipitation at various timescales (Table 1), we fitted distributed lag quasi-Poisson log-linear models to county-level incidence data at annual and weekly resolutions for all years in which both incidence and hydroclimatic data were available (2004–2012). Due to the highly clustered nature of leptospirosis incidence in time and space (Fig 1A and 1D), only counties reporting at least one case during the study period (99 counties of 181 counties in Sichuan) were included in regression analyses, and only weeks within the time period from August to October, when leptospirosis cases occurred, were included in the weekly model. This model formulation results in a distribution of counts of cases with a lower bound of 0, when no case occurred in that county in a given year or week. Quasi-Poisson regression was chosen to accommodate potential overdispersion in the count data.

Expected counts of cases y_i,t_ in county i and time t were modeled in relation to rainfall, soil moisture, and minimum temperature at multiple lags via a logarithmic link function:

$$logE[y_{i,t}]=\log\left(N_{i,t}\right)+{\beta_{0}}_{i}+\sum_{l=0}^{l_{max}}\beta_{l}X_{i,t-l}$$ (1)

where log(N_i,t_) is an offset accounting for the total population in county *i* at time t; ${\beta_{0}}_{i}$ is a county-specific fixed effect accounting for heterogeneity in average risks across counties; and the effects of environmental predictors **X**_i,t−l_ are estimated at all relevant lags *l* via coefficients *β*_*l*_. Following literature review regarding lags along pathways linking precipitation and leptospirosis transmission, and preliminary cross-correlation analyses between precipitation and leptospirosis cases, the maximum lag, l_max_, was set to one year in the yearly model, and to 15 weeks in the weekly model. Additionally, in the weekly model, we included a cubic regression spline with two degrees of freedom on study week to control for variable seasonality in disease risks, constrained so that the time spline is zero before and after each incidence season. This provided a level of flexibility sufficient to capture variability in the timing and magnitude of incidence across transmission seasons. During model construction, covariates of interest were screened based on pairwise correlation coefficients and generalized inflation factors [41] to determine which sets of covariates yield interpretable parameter estimates without excessive multicollinearity when included in the same model (S1 Text).

Results are presented as incidence rate ratios (IRRs) and associated 95% confidence intervals (CIs) corresponding to an increase in exposure equivalent to the variable’s interquartile range. IRRs and CIs were estimated from the quasi-Poisson regression using a robust sandwich estimator [42,43]. Cumulative effects of hydroclimatic exposures over all lags examined were calculated using the package dlnm [44]. To examine the potential for soil moisture to mediate precipitation’s influence on leptospirosis incidence, we applied the difference method and examined changes in regression parameters that would be indicative of mediation [45].

## Results

### Incidence and fatality rate

From January 2004 through December 2014, 2,934 leptospirosis cases in Sichuan were reported to China’s National Infectious Disease Reporting System (NIDRS), with 9% suspected, 84% clinically diagnosed, and 7% laboratory confirmed cases. Overall, this corresponds to an average annual incidence of 0.36 cases per 100,000 population. Among the reported cases, 41 were fatal (1.4% case fatality rate).

### Demographic features of reported cases

**Fig 2:**
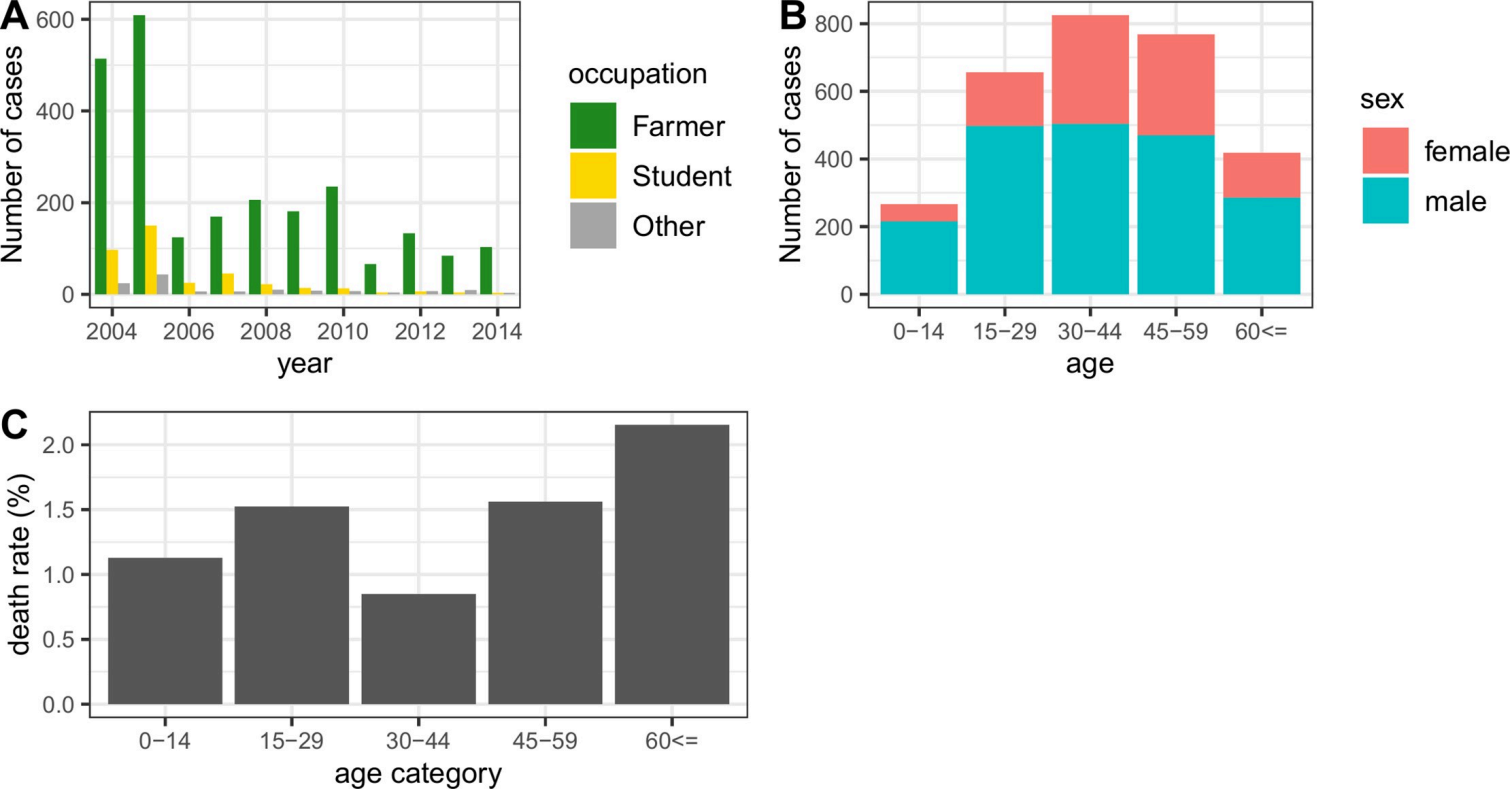
Number of leptospirosis cases reported in Sichuan, China, 2004–2014. A: Annual case counts aggregated by occupation. B: Case counts aggregated by age and sex. C: Mortality rate by age category.

Among all leptospirosis cases, the predominant occupations were farmers (83%) and students (13%), with the proportion of students gradually decreasing, particularly after 2010 (Fig 2A). The male-to-female ratio among cases (2.06:1 across all years) is higher than the male-to-female ratio of Sichuan’s population (1.16:1) [28]. All age groups are affected by leptospirosis (Fig 2B), and the mortality rate is highest among individuals aged >60 (2.1%) and lowest for individuals aged between 30 and 44 (0.8%) (Fig 2C).

### Spatiotemporal distribution of incidence

Leptospirosis incidence is concentrated in the eastern part of Sichuan, with most cases occurring within two high-risk areas as identified by the cluster analysis (Fig 1A). The first high-risk area is localized within one county in the northeast (Yilong county; 537 cases). The second high-risk area is a larger region overlapping multiple prefectures in the southeast (Ya’an, Meishan, Leshan, Ziyang, Neijiang, Zigong and Yibin prefectures; 1252 cases). Leptospirosis incidence decreased nonlinearly over the study period (Fig 1B). Incidence was highest in 2004–2005, with a mean incidence of 0.083 cases per year per 100,000 population, which dropped to 0.0241 in 2006–2010, and further to 0.0117 in 2011–2014. Mean yearly leptospirosis incidence rates were about 10 times higher in the northeastern and southeastern clusters than in the province as a whole. Yearly incidence rates decreased in the southern cluster but exhibited no clear downward trend in the northern cluster (Fig 1C). Case reports exhibit pronounced seasonality (Fig 1D), with 97% of all cases occurring between August and October (13% August, 75% September, 9% October). Peak incidence rates for the province always occurred in September (Fig 1E). The cubic spline regression estimated the average yearly timing of the peak to be September 12 (earliest peak date: September 4; latest peak date: September 22), with a mean duration of transmission of 79 days (standard deviation ±20 days; shortest: 53 days; longest: 115 days).

**Fig 3:**
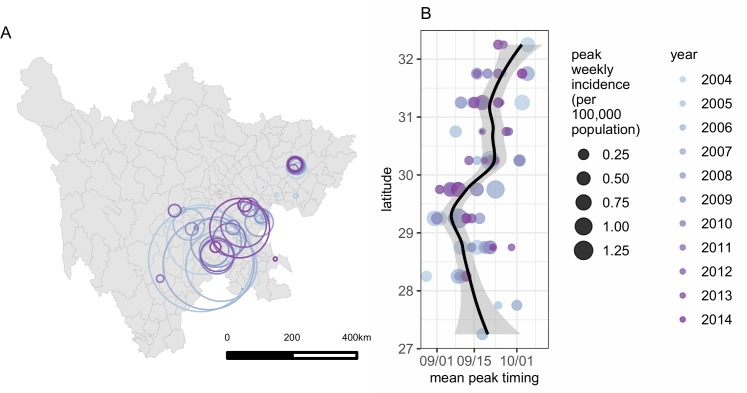
Annual and seasonal spatiotemporal patterns of leptospirosis incidence in Sichuan, China. A: Spatial distribution of yearly high incidence clusters of leptospirosis. Clusters were detected by running the Poisson scan statistic with SaTScan separately for every year. This map was generated using data provided by Sichuan CDC for the purpose of this study, and plotted in R statistical software using ggplot2. B: Latitudinal gradient in the seasonal timing of peak leptospirosis incidence. Dots are yearly values of peak timing per latitude band, their size corresponds to the amplitude of the seasonal peak in leptospirosis incidence. The line is the estimated smoothed timing of latitudinal peaks weighted by peak amplitudes, with estimated 95% CI shaded.

Yearly cluster analyses detected a northern high-risk cluster of nearly identical extent every year, consistently located near Yilong county (Fig 3A). In contrast, the extent and location of high-risk clusters in the southern region varied over the study period, with an apparent decrease in spatial extent and northward shift over time. Timing of leptospirosis seasonality varied over 0.5° latitude bands; peak incidence occurred earlier in the mid-latitudes of the southern region (mean: September 5) than in the northern region (mean: September 20) (Fig 3B).

### Associations with hydroclimatic variables at inter- and intra-annual timescales

The association between hydroclimatic risk factors and leptospirosis incidence was assessed using statistical modeling, where hydroclimatic risk factors included variables of a large-scale hydrological model (see Methods section). Results are expressed as incidence rate ratios (IRRs) corresponding to the discrete effect over a given lag of an interquartile range (25th to 75th percentile) increase in each hydroclimatic exposure, as estimated by a distributed lag quasi-Poisson regression model. Reference values for hydroclimatic exposure variables, as well as associated time lags are listed in Table 2, hydroclimatic variables considered in the models are defined in Table 3 and Table 4 (see S1 Text for information supporting model variable selection). Controlling for county fixed effects, models including single hydroclimatic variables at 0–1 year lags revealed positive associations for annual precipitation (lag 0 IRR: 2.01; 95% CI 1.70–2.39; lag 1 IRR: 1.72; 95% CI 1.44–2.07) and soil moisture (lag 0 IRR: 4.67; 95% CI 3.77–5.81; lag 1 IRR: 1.95; 95% CI 1.59–2.38). Annual mean temperature was collinear with county fixed effects, and we therefore excluded temperature from the yearly regression analyses presented here (see S1 Text for analyses of multicollinearity, and S1 Table for results including mean minimum temperature). When annual precipitation and soil moisture were incorporated in the same model, precipitation IRRs shifted towards the null (lag 0 IRR: 0.99; 95% CI 0.79–1.23; lag 1 IRR: 1.06; 95% CI 0.87–1.30), while IRRs associated with increasing soil moisture remained elevated (lag 0 IRR: 4.76; 95% CI 3.55–6.43; lag 1 IRR: 1.86; 95% CI 1.46–2.37). Model fits incorporating annual runoff showed no evidence of association with leptospirosis incidence (lag 0 IRR: 1.10; 95% CI 0.95–1.27; lag 1 IRR: 1.02; 95% CI 0.87–1.19) (S1 Table).

**Table 3:** Results of regression analyses of hydroclimatic risk factors for human leptospirosis incidence at the yearly timescale and county resolution. Incidence rate ratios (IRR) and 95% confidence intervals (CIs), were estimated using quasi-Poisson regression and the robust sandwich estimator for variance [42,43] and correspond to an increase in exposure equivalent to the exposures interquartile range within the dataset (0.67 mm precipitation (P); 1.19 mm soil moisture (θ)). Reference values for the each of the exposure variables are presented in Table 2. Bolded values correspond to associations that are statistically significant at the 95% confidence level. Each row corresponds to one model fit. Information supporting variable selection can be found in S1 Text. Results for regressions including other hydroclimatic predictors are presented in S1 Table.

Hydroclimatic exposures in model*	${IRR}_{P_{t}}^{†‡}$ (95% CI)	${IRR}_{P_{t-1}}$ (95% CI)	IRR_θ,t_ (95% CI)	IRR_θ,t−1_ (95% CI)
P_t−1:t_	**2.01** **(1.70–2.39)**	**1.72** **(1.44–2.06)**	-	-
θ_t−1:t_	-	-	**4.67** **(3.77–5.81)**	**1.95** **(1.59–2.38)**
P_t−1:t_+ θ_t−1:t_	0.99 (0.79–1.23)	1.06 (0.87–1.30)	**4.76** **(3.55–6.43)**	**1.86** **(1.46–2.37)**

Abbreviations and symbols: IRR–incidence rate ratio; CI–confidence interval; T_min_–mean minimum daily temperature; P–mean daily precipitation; θ–mean daily water content in top 10 cm of soil

*Regressions included the indicated hydroclimatic exposures as well as county fixed effects to control for long-term differences in baseline risk

†Incidence rate ratios correspond to an increase in hydroclimatic exposures equivalent to the difference between the 25th to 75th percentile of values observed during the study period

‡Subscripts *t* and *t*−1 correspond to exposures at zero- and one-year lags, respectively

**Table 4:** Results of regression analyses of hydroclimatic risk factors for human leptospirosis incidence at the weekly timescale and county resolution, encompassing the transmission season of leptospirosis in Sichuan (August-October). Incidence rate ratios (IRR) and 95% confidence intervals (CIs), were estimated using quasi-Poisson regression and the robust sandwich estimator for variance and correspond to an increase in exposure equivalent to the interquartile range of the variable (4.36 mm precipitation (P); 3.39 mm soil moisture (θ)). Reference values for the each of the exposure variables are presented in Table 2. Bolded values correspond to associations that are statistically significant at the 95% confidence level. Information supporting variable selection can be found in S1 Text. Results for regression including other hydroclimatic predictors are included in S2 Table.

Lag (in weeks)	Hydroclimatic exposures: P	Hydroclimatic exposures: P+θ	
	${IRR}_{P}^{†}$ (95% CI)	IRR_P_ (95% CI)	IRR_θ_ (95% CI)
0	0.94 (0.85;1.04)	0.92 (0.79;1.06)	1.06 (0.89;1.26)
1	1.04 (0.95;1.12)	0.98 (0.86;1.10)	1.12 (0.95;1.33)
2	**1.12** **(1.04;1.20)**	1.03 (0.93;1.14)	**1.19** **(1.00;1.43)**
3	**1.13** **(1.06;1.21)**	1.07 (0.97;1.18)	1.11 (0.93;1.32)
4	**1.10** **(1.03;1.18)**	1.08 (0.98;1.19)	1.04 (0.87;1.24)
5	**1.08** **(1.01;1.15)**	1.08 (0.98;1.18)	0.98 (0.83;1.18)
6	**1.10** **(1.03;1.17)**	1.05 (0.95;1.14)	1.16 (0.96;1.40)
7	**1.07** **(1.00;1.14)**	0.99 (0.90;1.09)	1.20 (0.99;1.46)
8	1.06 (0.99;1.12)	1.04 (0.94;1.14)	0.99 (0.82;1.20)
9	0.99 (0.93;1.06)	1.00 (0.90;1.10)	0.99 (0.82;1.20)
10	1.06 (0.99;1.12)	1.06 (0.96;1.16)	1.00 (0.83;1.21)
11	**1.08** **(1.01;1.16)**	1.02 (0.92;1.13)	1.17 (0.98;1.42)
12	1.07 (0.99;1.16)	1.01 (0.90;1.14)	1.12 (0.93;1.35)
13	1.01 (0.92;1.11)	1.01 (0.88;1.16)	0.97 (0.81;1.18)
14	0.96 (0.86;1.06)	0.98 (0.83;1.16)	0.92 (0.76;1.13)
15	0.89 (0.79;1.01)	1.01 (0.83;1.22)	0.83 (0.68;1.02)

Abbreviations and symbols: IRR–incidence rate ratio; CI–confidence interval; P–mean daily precipitation; θ–mean daily water content in top 10 cm of soil

†Incidence rate ratios correspond to an increase in hydroclimatic exposures equivalent to the difference between the 25th to 75th percentile of values observed during the study period.

Cumulative IRRs correspond to the cumulative effect over all lags of an interquartile range increase in each hydroclimatic exposure in the distributed lag quasi-Poisson regression model. Cumulative IRR associated with interquartile range increase in precipitation for the current and prior year was 3.45 (2.57–4.64) when not controlling for soil moisture; cumulative IRRs associated with interquartile range increases in precipitation and soil moisture for the current and prior years in the full yearly model were 1.05 (0.74–1.49) and 8.84 (6.23–12.52), respectively. The change in the effect estimate of precipitation between models excluding and including soil moisture—from 3.45 (2.57–4.64) to 1.05 (0.74–1.49)—is indicative of mediation of the effect of precipitation by soil moisture at the yearly time scale [45].

At the weekly timescale, we report lags for which the association between a hydroclimatic variable and leptospirosis risk was significant, as well as the lag for which the IRR was the largest. At the weekly timescale, a model with weekly precipitation revealed evidence of elevated leptospirosis risk with increasing precipitation at 2–7 and 11 week lags (largest IRR at 3 week lag: 1.13; 95% CI 1.06–1.21) (Table 4). Adding weekly soil moisture to the model revealed that increased soil moisture was significantly associated with increasing leptospirosis incidence at a 2 week lag (2 week lag: 1.19; 95% CI 1.00–1.43), while precipitation effects shrank toward the null (largest IRR at 4 week lag: 1.03; 95% CI 0.93–1.14). Cumulative IRR associated with an interquartile range increase in precipitation at 0–15 week lags was 1.90 (1.18–3.06) when not controlling for soil moisture; cumulative IRRs associated with interquartile range increases in precipitation and soil moisture at 0–15 week lags in the full weekly model were 1.36 (0.72–2.57) and 2.13 (0.97–4.68), respectively. Model fits incorporating weekly runoff showed evidence of a modest association with leptospirosis incidence (largest IRR at 3 week lag: 1.04; 95% CI 1.01–1.07) (S2 Table). Sensitivity analyses showed consistency of associations under varying formulations of the time spline, while omission of the time spline resulted in hydroclimatic associations at more and longer lags (2–11 weeks for precipitation, 1–8 weeks for soil moisture). The change in the effect estimate of precipitation between models excluding and including soil moisture—from 1.90 (1.18–3.06) to 1.36 (0.72–2.57)—is indicative of mediation of the effect of precipitation by soil moisture at the weekly time scale [45].

## Discussion

The present study investigated demographic and spatiotemporal properties of leptospirosis incidence and its association with hydroclimatic risk factors in a high-risk region in western China. From January 2004 to December 2014, 2,934 leptospirosis cases were reported in the study region, accounting for 35.6% of China’s total number of leptospirosis cases (8,238) [46]. The average annual incidence rate in the region of 0.36 cases per 100,000 population is substantially higher than China’s overall incidence rate for 2001–2010 (0.11 cases per 100,000) [26].

Leptospirosis cases occurred exclusively in the eastern part of the province, which consists of low-lying fertile plains, and where most of the population is engaged in agriculture. Demographic features of reported cases are aligned with findings from previous studies: cases are predominantly farmers and the male-to-female ratio is higher within leptospirosis cases (2.06:1 across all years) than within Sichuan’s general population (1.16:1) [28], indicating higher risk of transmission among men. This pattern has previously been explained by exposures within occupations that more commonly involve males, such as types of agricultural work that involve contacting water [3,26].

Sichuan shared in China’s long-term trend towards decreasing leptospirosis incidence [26,47]. A number of explanations have been put forward to explain the long-term decrease in reported leptospirosis over time in China, including improvements in sanitation, vaccination campaigns of high-risk populations [48], mechanization of agriculture, and the inclusion of antibiotics active against leptospires in commercial feeds for captive-bred pigs [47,49]. Examination of reported cases in Sichuan extending back to 1990 reveals a continual downward trend in leptospirosis which the 2004–2005 data seem to align with (S2 Text), thus it does not appear that 2004–2005 were abnormal years, either with respect to surveillance (the NIDRS was established in 2004), or disease occurrence. We also noted that leptospirosis incidence sharply decreased in years 2006 and 2011, during which parts of Sichuan experienced extreme drought conditions [50,51].

Leptospirosis cases are highly clustered spatially, temporally and occupationally in Sichuan: spatial scan statistics revealed two areas of the province in which most cases occurred; nearly all reported cases occur in the same 5-week period from late August to early October every year; and a great majority and increasing proportion of cases over the period occurred among farmers. Notably, while incidence rates declined substantially (and shifted geographically) within the southern high-risk region, leptospirosis in the northern high-risk region did not exhibit a downward trend over the study period. The reasons for this disparity are beyond the scope of the present analysis but remain a topic of interest for later work.

Seasonal clustering of leptospirosis cases has been described in other parts of the world [17], but the seasonal pattern we observe in the study region is remarkable for its stability and magnitude, with nearly all cases occurring within a five week period each year. The consistency of timing of leptospirosis incidence would likely be incompatible with natural events, such as flooding. We are not aware of any regular annual flooding in Sichuan that consistently occurs between August and October. Regression analyses found no association between runoff and incidence at a yearly timescale, and a modest association at the weekly time scale (S1 Table and S2 Table), and the time series of runoff data from the VIC model suggests that major runoff events occur throughout much of the year, and are not consistently associated with the leptospirosis incidence season (S3 Text). Instead, it seems likely that scheduled human behaviors influencing exposure determine the timing of human leptospirosis in the study region, rice harvesting being a plausible candidate [52]. In Sichuan, rice harvest timing ranges between mid-August to mid-September, with a geographical gradient from Southern to Northern regions and from low to high elevations (Quanzhong Ge, Sichuan Department of Agriculture, pers. comm.). This is consistent with the observation that leptospirosis peaks earlier in southern latitudes when compared to northern latitudes (Fig 3B). Furthermore, a recent retrospective case-control study in Yilong county identified the presence of standing water during rice harvest as the strongest predictor of leptospirosis occurrence [52], further suggesting the role of occupational exposures associated with rice harvest.

Regression analyses revealed positive associations between precipitation and incidence at multiple timescales: at a yearly resolution, leptospirosis was associated with increased precipitation in the current or previous year; at a weekly resolution, leptospirosis was associated with increased precipitation 2–7 weeks prior to the reporting of a case (or 0–6 weeks prior to exposure, accounting for the typical incubation period of 7–12 days) [3]. Associations at every timescale appear to be mediated through surface soil moisture. These findings suggest that leptospirosis may be driven by seasonal agricultural activity is Sichuan, and that moist soils in time periods preceding harvest may increase disease risks from weekly to multi-yearly time scales.

These results suggest that the primary mechanisms through which precipitation influences leptospirosis in Sichuan may be related to ecological processes impacting pathogen survival in moist soils or host abundance. Changes in soil moisture can exert a multi-year influence on rodent abundance by affecting food availability [9,13]. Longitudinal data on mean rodent abundance across several monitored sites in Sichuan provided by the national sentinel surveillance system for leptospirosis (S4 Text) indicate that rodent populations are decreasing over the study period, suggesting a potential role of host population changes in driving the observed decrease in leptospirosis incidence. At weekly to yearly time scales, increased soil moisture may act to facilitate survival and accumulation of pathogenic leptospires in soil and water [14,21,22,53–55]. Another possible mechanism at weekly lags is the displacement of rodent hosts from their burrows as soil water tables rise, driving them into closer proximity with human populations [3,26,56]; however, we would expect such saturated conditions to be correlated with increased runoff.

The duration of pathogenic *Leptospira* spp. survival above the infectious dose in humans is poorly understood [14]. Some studies report long survival times, with mean time to extinction of 130 days in culture experiments at 4°C, 263 days at 20°C, and 316 days at 30°C in fresh water [15,16]. Others suggest survival over shorter time periods, with culture experiments in soils indicating *Leptospira* spp. survival of 2–7 weeks [17], or 2–4 weeks, with the limitation that long-term persistence seems to occur at concentrations close to or below the experimental limit of detection [18]. *Leptospira* spp. may survive and remain virulent for months even in cold conditions, as well as in nutrient-poor, acidic conditions [15,16].

Our findings regarding the timescale of associations of leptospirosis and precipitation are consistent with those reported in similar studies in rural settings [7,8,22,57]. On the other hand, we found an association between leptospirosis incidence and wet soils, when prior studies reported associations with indicators for flooding [3,19,26,56]. This would seem to indicate that exposures are linked to occupational activities, rather than during extreme events in the study region, with increasing risk as conditions for pathogenic *Leptospira* spp. survival in soils are favorable (moist soils, possibly high temperatures). These results are consistent with previous observations indicating that soils are environmental reservoirs playing a critical role in the transmission of pathogenic *Leptospira* spp. [58].

Limitations of this work arise due to the relatively coarse spatial resolution of the hydrological data, as well as the complex nature of the disease system under study. The hydrological model used to estimate soil moisture and runoff exposures can only represent spatially averaged values of hydrological outcomes at 0.25° (~28 km) resolution; sub-grid spatial heterogeneity can lead to differential hydrology across the land surface, which may result in some misclassification of exposures within each grid cell. Due to the non-specific, flu-like symptoms of mild leptospirosis cases, many infections may not be reported, or may be misdiagnosed. The extreme, nearly binary seasonality of leptospirosis observed in this study complicates analysis at the weekly resolution, increasing the likelihood of unresolvable confounding between observed variables of interest and unmeasured drivers of seasonality, such as rice harvesting. We were able to control for average seasonal patterns through the inclusion of spline terms, but there may still be residual confounding by unobserved variables. Another limitation in this study is that most cases were suspected and clinically diagnosed, and only 7% of the cases were laboratory confirmed. Further research may elucidate the causes of persistent risk in the northern cluster, or refine our understanding of environmental and ecological risk factors with higher-resolution hydrological predictions, land use data, or data regarding the environmental abundance of pathogenic *Leptospira* spp. and intermediate hosts in the environment.

Our work presents an unusual and intriguingly seasonal leptospirosis dataset and contributes novel use of hydrological model outputs at multiple timescales to disentangle the complex environmental pathways driving incidence. Our findings suggest that the survival of *Leptospira* spp. in moist soils may be a critical control on leptospirosis transmission during the autumn rice harvest in Sichuan province. Better understanding regional environmental and social drivers of infection in Sichuan will allow health practitioners and policy-makers to develop effective prevention programs, build capacity in emergency response and reduce disease burden. This research was carried out in a close collaboration between university researchers and the China Centers for Disease Control and Prevention, and the research results have important potential for translation into targeted public health actions and policies. For one, the findings identify the geographic foci for leptospirosis prevention in Sichuan—the two endemic regions discussed herein, and the intense nature of the leptospirosis transmission season in late summer and early fall. The results indicate the particular importance of leptospirosis prevention in years of elevated precipitation, and particularly in areas where soil saturation is high. During these periods in particular, prevention of leptospirosis might emphasize occupational protections (e.g, protective equipment when working near potentially contaminated water or wet soil; covering open wounds with waterproof bandages; etc.). Additionally, the research presented here can be valuable for understanding the potential for climate change to alter the risk of leptospirosis transmission in future decades. Such projections can provide critical guidance for long-term planning and for targeting control and prevention activities. What is more, results from the time-series analyses presented here can be useful to establish earlier warning of seasonal onset, as well as a basis for monitoring changes in seasonal transmission as agricultural systems change in China’s highly dynamic economy. These areas are topics of ongoing and future research for our group, and we hope, for others.

## Supporting information

S1 Text Investigation of collinearity using pairwise correlation coefficients and variance inflation factors. (DOCX)

S2 Text Number of reported leptospirosis cases in the Sichuan province, 1990–2014. (DOCX)

S3 Text Weekly county-level leptospirosis incidence rates and weekly mean runoff rates at the county-level during the study period. (DOCX)

S4 Text Yearly rodent density and yearly soil moisture. (DOCX)

S5 Text STROBE Checklist for observational studies. (DOC)

S1 Table Supplementary results of regression analyses of hydroclimatic risk factors for human leptospirosis incidence at the yearly timescale and county resolution. (XLSX)

S2 Table Supplementary results of regression analyses of hydroclimatic risk factors for human leptospirosis incidence at the weekly timescale and county resolution. (XLSX)
