# Supplementary material for: Hydroclimatic drivers of highly seasonal leptospirosis incidence suggest prominent soil reservoir of pathogenic Leptospira spp. in rural western China
Source: PLoS Negl Trop Dis. 2019 Dec 26;13(12):e0007968. doi: 10.1371/journal.pntd.0007968 (PMC6948824; doi:10.1371/journal.pntd.0007968)
Supplement: S1 Text — (DOCX) [file pntd.0007968.s001.docx]

Appendix

Investigation of collinearity using pairwise correlation coefficients and variance inflation factors

[1 Introduction 1](#_Toc15822641)

[2 Method 1](#_Toc15822642)

[3 Results and Discussion 2](#_Toc15822643)

[3.1 Yearly regression models 2](#_Toc15822644)

[3.1.1 Pairwise correlation coefficients 2](#_Toc15822645)

[3.1.2 Variance inflation factors 2](#_Toc15822646)

[3.2 Weekly regression models 3](#_Toc15822647)

[3.2.1 Pairwise correlation coefficients 3](#_Toc15822648)

[3.2.2 Variance inflation factors 4](#_Toc15822649)

[4 Conclusion 4](#_Toc15822650)

[5 References 4](#_Toc15822651)

# Introduction

The analysis presented in the main text uses statistical regression to investigate relationships between hydroclimatic risk factors and leptospirosis in China. A particular focus is to explore the use of soil moisture and surface runoff as mediating variables to disentangle pathways linking rainfall to leptospirosis incidence.

While some level of collinearity between the exposure variable and the potential mediating variable is desired in order to be in a position to apply a mediation analysis (1), elevated collinearity increases the variance of the estimates of the regression coefficients, and can increase the risk of spurious findings in some circumstances (2,3). This appendix identifies and addresses problems caused by collinearity between hydroclimatic variables, and supports decisions regarding variables to be included in regression models presented in the main text.

# Method

Two measures were used to assess the inclusion of variables in regression models: pairwise correlation coefficients and variance inflation factors (VIFs).

We first screened groups of variables based on their pairwise correlation coefficient by computing the correlation matrix of the predictors. If a correlation coefficient indicated that two predictors were too closely related to each other, we excluded one of the variables from regression models. We used a threshold of 0.8 to determine that the correlation between two predictors was too high for the predictors to be included in the same model.

We then assessed problems caused by multicollinearity by computing variance inflation factors (VIFs) in the context of the regression of interest (Eq. 1 in the main text). VIFs measure the increase in variance of an estimated regression coefficient due to collinearity, indicating how much larger the variance is compared with what it would be if the corresponding variable were uncorrelated with the other predictor variables in the model (4). A high VIF associated with a predictor variable indicates high collinearity with other predictor variables in the model, and suggests that regression estimates associated with that predictor variable should not be interpreted, or alternatively that the predictor variable should be removed from the regression model in order to avoid unstable effect estimates. Depending on sources, it is recommended for VIF values to be lower than 5 (5) or 10 (6).

For terms having more than one degree of freedom (the lagged terms), we calculate generalized variance-inflation factors (GVIF) (7). GVIFs indicate how much larger the confidence volume is for the corresponding subset of coefficients compared with what it would be if the regressors in this subset were uncorrelated with regressors in the complementary subset. In order to compare the generalized variance-inflation factor values to the usual thresholds, we reported values adjusting for the dimensionality df of that subset by calculating GVIF^1/df^, as recommended in (7).

VIF values were estimated by applying the vif function from the car R package to outputs of the distributed-lag quasi-Poisson log-linear models (8).

# Results and Discussion

## Yearly regression models

### Pairwise correlation coefficients

Pairwise correlation coefficients between hydroclimatic predictors are included in Figure 1, where the color indicates the strength of the pairwise correlation coefficients. Correlation coefficients are greater than 0.9 for pairs $(P_{t},Q_{t})$, $\left( P_{t-1},Q_{t-1} \right)$, and $(T_{min, t-1}, T_{min, t})$. We therefore excluded regression models with both precipitation and runoff, and excluded $T_{min, t-1}$ from all regression models.


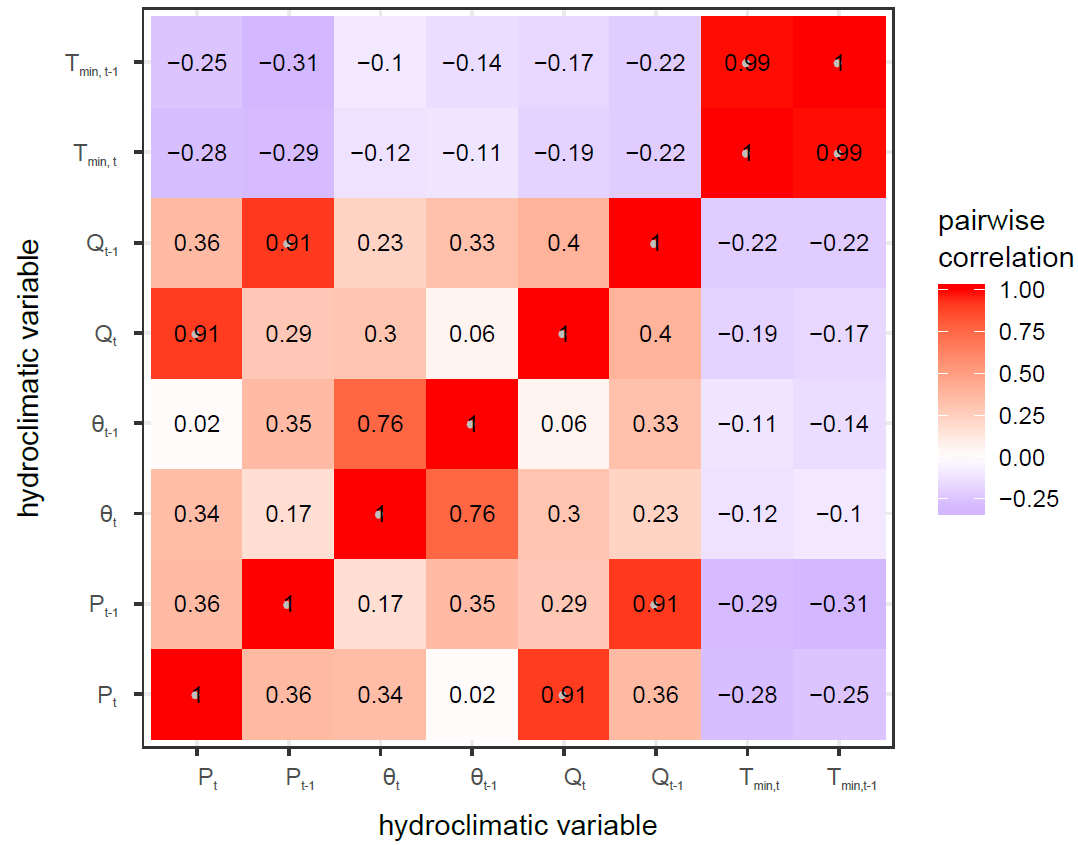


Figure 1 – Correlation matrix of hydroclimatic variables at the yearly timescale. The color indicates the strength of the correlation between two variables.

### Variance inflation factors

Table 1 reports GVIF^1/df^, the generalized variance-inflation factor values adjusted for the number of dimensions. The tables follow the presentation of regression analyses results in the main text, where each row corresponds to a different set of predictors in the statistical model. There is one value by hydroclimatic variable because lagged variables are grouped together.

In all models, variance inflation factors associated with temperature variables are elevated, even when the only predictors in the model are yearly mean temperature and county-level fixed effects. This suggests that annual temperature and county-level fixed effects are strongly associated, where variations in temperature are more important between counties in a given year than between years in a given county.

For all models without a temperature component, generalized variance-inflation factors are lower than 5. We present regression results for these models in the main text.

Table 1 - Generalized variance-inflation factor values GVIF^1/df^ in yearly statistical models, adjusted for the number of dimensions. Values above 5 (or 10) indicate high collinearity with regressors in the complementary subset and suggest that regressors should not be included in the same model. We colored generalized variance-inflation factor values in green if they correspond to reasonable values (GVIF^1/df^ < 5), in orange if they are reasonable depending on the source (5 < GVIF^1/df^ < 10), and in red if they are too high for the corresponding predictor to be included in the same model (GVIF^1/df^ > 10).

| **Model** | **GVIF^1/df^** | | | | **Included in main text** | **Included in Supporting Information** |
| --- | --- | --- | --- | --- | --- | --- |
|  | **P** | **Q** | $\boldsymbol{\theta}$ | **T** |  | ***** |
| **(ys1)** |  |  |  | 76.7 |  | * |
| **(ys2)** | 2.5 |  |  | 101.4 |  | * |
| **(ys3)** |  | 1.9 |  | 80 |  | * |
| **(ys4)** |  |  | 3.5 | 182.1 |  | * |
| **(ys5)** | 3.7 |  | 5.2 | 184.7 |  | * |
| **(y1)** | 2.3 |  |  |  | * |  |
| **(ys6)** |  | 1.8 |  |  |  | * |
| **(y2)** |  |  | 2.5 |  | * |  |
| **(y3)** | 3.7 |  | 4.1 |  | * |  |

## Weekly regression models

### Pairwise correlation coefficients

Given the number of investigated lags in the weekly models, we calculated the correlation matrix for non-lagged variables, and evaluated correlation in time by computing autocorrelograms separately for each variable.

Figure 2 presents pairwise correlation coefficients for non-lagged hydroclimatic variables at the weekly timescale. The correlation coefficient is greater than 0.9 for precipitation and surface runoff. We therefore excluded models with both precipitation and runoff.


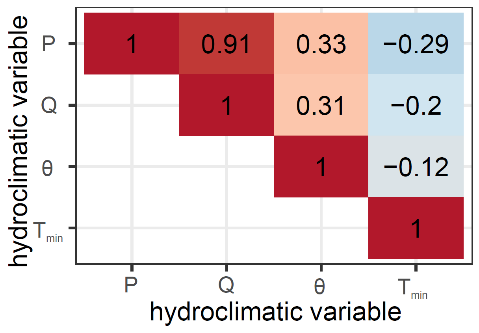


Figure 2 - Correlation matrix of hydroclimatic variables at the weekly timescale. The color indicates the strength of the correlation between two variables.

Figure 3 presents values of autocorrelation coefficients for weekly timeseries of hydroclimatic factors. Autocorrelation values are lower than the confidence level for precipitation and soil moisture; however temperature is highly autocorrelated. In order to avoid unstable regression coefficients due to high autocorrelation in temperature, we included temperature at lag 2 weeks only in the regression rather than using the entire temperature timeseries, where 2 weeks is a typical duration between exposure and the onset of symptoms.


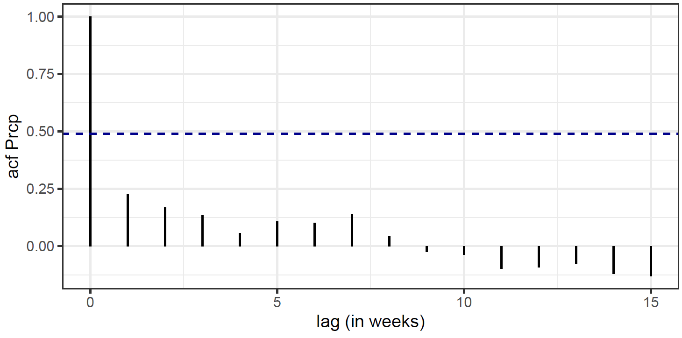
.
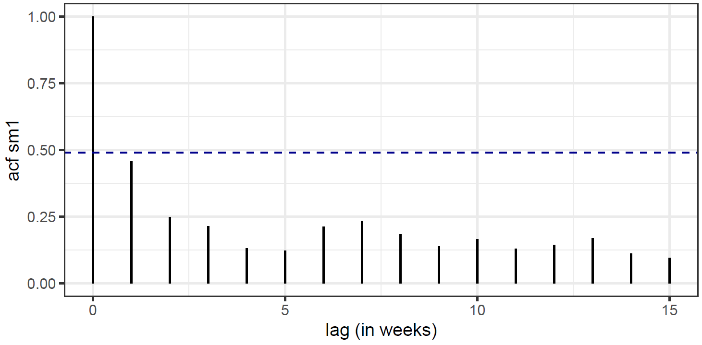


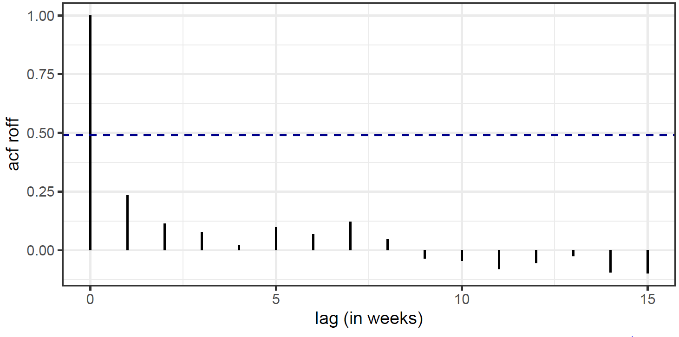

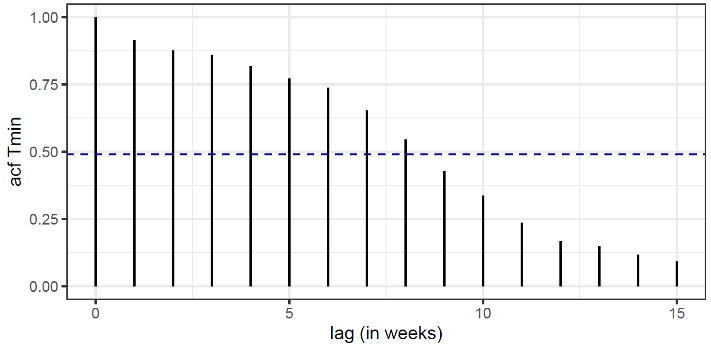


Figure 3 – Autocorrelation function for hydroclimatic factors (top left: precipitation; top right: soil moisture; bottom left: surface runoff, bottom right: temperature). The height of each bar indicates the strength of the correlation between any two lags. The horizontal dashed line is the confidence interval band.

### Variance inflation factors

Table 2 presents the generalized variance-inflation factors for the weekly models. Similarly, variance inflation factors associated with temperature variables are elevated, suggesting a strong collinearity between temperature at lag 2 weeks and county-level fixed effects.

For all models without a temperature component (w4-w5), generalized variance-inflation factors are lower than 5. We present regression results for these models in the main text.

Table 2 - Generalized variance-inflation factor values GVIF^1/df^ in weekly statistical models, adjusted for the number of dimensions. Values above 5 (or 10) indicate high collinearity with regressors in the complementary subset and suggest that regressors should not be included in the same model. We colored generalized variance-inflation factor values in green if they correspond to reasonable values (GVIF^1/df^ < 5), in orange if they are reasonable depending on the source (5 < GVIF^1/df^ < 10), and in red if they are too high for the corresponding predictor to be included in the same model (GVIF^1/df^ > 10).

| **Model** | **GVIF^1/df^** | | | | **Included in main text** | **Included in supporting information** |
| --- | --- | --- | --- | --- | --- | --- |
|  | **P** | **Q** | $\boldsymbol{\theta}$ | **T** |  |  |
| **(ws1)** |  |  |  | 7.11 |  | * |
| **(ws2)** | 1.28 |  |  | 8.83 |  | * |
| **(ws3)** |  | 1.22 |  | 8.51 |  | * |
| **(ws4)** |  |  | 1.38 | 9.12 |  | * |
| **(ws3)** | 2.7 |  | 2.9 | 11.6 |  | * |
| **(w1)** | 1.26 |  |  |  | * |  |
| **(ws6)** |  | 1.21 |  |  |  | * |
| **(ws7)** |  |  | 1.36 |  |  | * |
| **(w2)** | 2.64 |  | 2.85 |  | * |  |

# Conclusion

In yearly regression models, pairwise correlation coefficients indicate that precipitation and runoff should not be included in the same models, and that lagged temperature should not be included. Variance-inflation factors indicate a high collinearity between temperature and other predictors, suggesting that temperature should not be included in the yearly model. When temperature is not included, variance-inflation factor values indicate that other hydroclimatic predictors (precipitation and soil moisture) can be included.

Similarly, in the weekly regression models, pairwise correlation coefficients indicate that precipitation and runoff should not be included in the same models, and autocorrelograms indicate that all lagged variables can be included except for temperature. Variance-inflation factor values indicate a high collinearity between temperature and other predictors, suggesting that temperature should not be included. When temperature is not included, variance-inflation factor values indicate that other hydroclimatic predictors (precipitation and soil moisture) can be included.

# References

1. VanderWeele TJ. Mediation Analysis: A Practitioner’s Guide. Annu Rev Public Health. 2016;37:17–32.

2. James G, Witten D, Hastie T, Tibshirani R. An Introduction to Statistical Learning. Vol. 112. Springer; 2013.

3. Yoo W, Mayberry R, Bae S, Singh K. A Study of Effects of MultiCollinearity in the Multivariable Analysis. 2015;17.

4. Fox J. Applied regression analysis and generalized linear models. Sage Publications; 2015.

5. Sheather S. A modern approach to regression with R. Springer Science & Business Media; 2009.

6. Hair JF, Black WC, Babin BJ, Anderson RE, Tatham RL. Multivariate data analysis (Vol. 6). Upper Saddle River, NJ: Pearson Prentice Hall; 2006.

7. Fox J, Monette G. Generalized Collinearity Diagnostics. J Am Stat Assoc. 1992;87(417):178–83.

8. Fox J, Weisberg S. An R Companion to Applied Regression [Internet]. Third. Sage; 2019. Available from: https://socialsciences.mcmaster.ca/jfox/Books/Companion/
