# Supplementary material for: Hydroclimatic drivers of highly seasonal leptospirosis incidence suggest prominent soil reservoir of pathogenic Leptospira spp. in rural western China
Source: PLoS Negl Trop Dis. 2019 Dec 26;13(12):e0007968. doi: 10.1371/journal.pntd.0007968 (PMC6948824; doi:10.1371/journal.pntd.0007968)
Supplement: S2 Text — (DOCX) [file pntd.0007968.s002.docx]

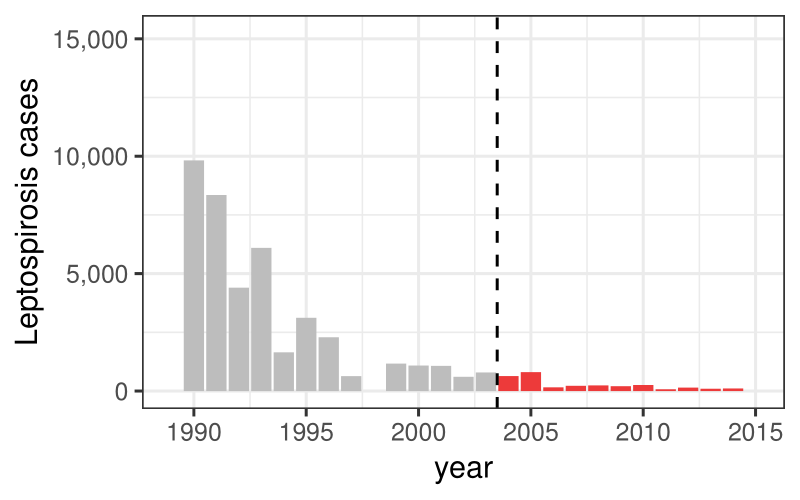


Number of reported leptospirosis cases in the Sichuan province, 1990-2014. The dashed line indicates a change in reporting system. Prior to 2004, cases were reported using the Public Health Information System (PHIS, in grey); starting from 2004, cases were reported via the National Infectious Disease Reporting System (NIDRS, in red). 1998 data is missing from the records.
