# Supplementary material for: Hydroclimatic drivers of highly seasonal leptospirosis incidence suggest prominent soil reservoir of pathogenic Leptospira spp. in rural western China
Source: PLoS Negl Trop Dis. 2019 Dec 26;13(12):e0007968. doi: 10.1371/journal.pntd.0007968 (PMC6948824; doi:10.1371/journal.pntd.0007968)
Supplement: S3 Text — (DOCX) [file pntd.0007968.s003.docx]

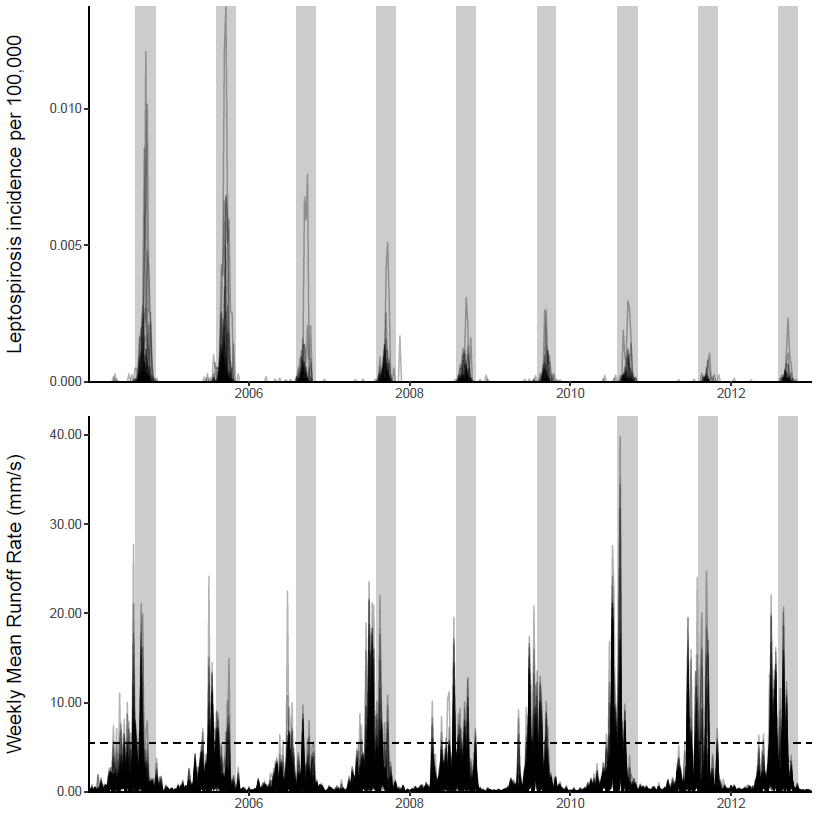


Upper panel shows weekly county-level leptospirosis incidence rates during the study period. Lower panel shows weekly mean runoff rates at the county-level. Shaded areas in both panels represent the transmission season considered during regression analyses, during which nearly all leptospirosis cases occur (August-October). Horizontal dashed line in lower panel represents the 97.5^th^ percentile of all runoff values observed during the study period.
