# Supplementary material for: Hydroclimatic drivers of highly seasonal leptospirosis incidence suggest prominent soil reservoir of pathogenic Leptospira spp. in rural western China
Source: PLoS Negl Trop Dis. 2019 Dec 26;13(12):e0007968. doi: 10.1371/journal.pntd.0007968 (PMC6948824; doi:10.1371/journal.pntd.0007968)
Supplement: S4 Text — (DOCX) [file pntd.0007968.s004.docx]

Yearly rodent density and yearly soil moisture

Figure 1 – Rodent density in Sichuan as provided by the National Leptospirosis Surveillance Program

The rodent time series represents the percent of traps with a rodent, i.e. the total number of traps that caught rodents over the total number of traps across all sites. The timeseries is aggregated across 11 sites in Sichuan (温江区、江油市、峨眉山市、名山县、邛崃市、东坡区和广安区，雅安市、达州市、宜宾市和凉山州). Each site corresponds to a prefecture or a county. Each site has at least 300 traps that were put each night over some period near farmland, streams, or other suspicious environments; however, the specific locations of the traps within each site is unknown. The surveillance is conducted twice each year - one in April to June, the other one in August to October.

The following figures could indicate a possible association between soil moisture, rodent density and leptospirosis incidence, although we are not drawing a conclusion because the datasets have a small size and are largely incomplete.


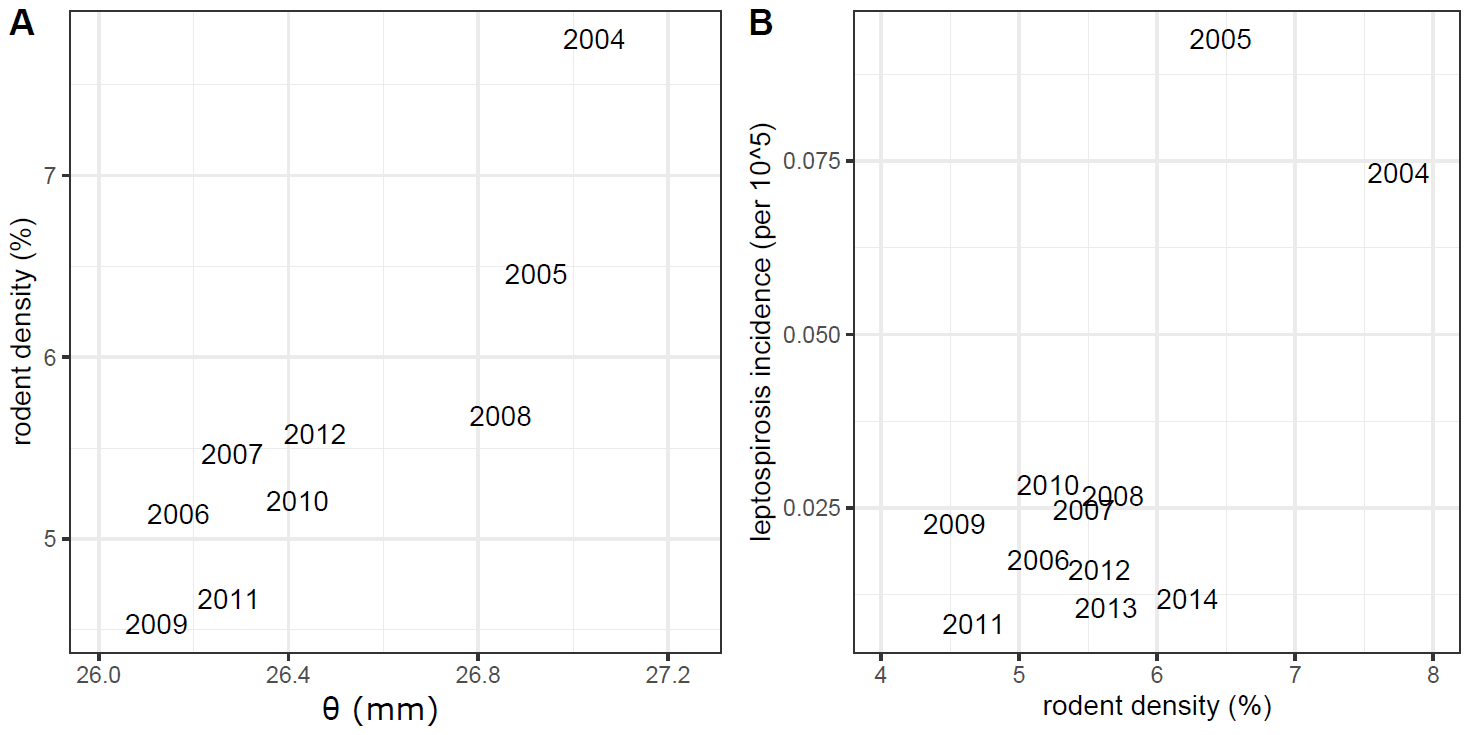


Figure 2 – (A) Yearly soil moisture and rodent density (B) Yearly rodent density and leptospirosis incidence in Sichuan
